# Injections, Cocktails and Diviners: Therapeutic Flexibility in the Context of Malaria Elimination and Drug Resistance in Northeast Cambodia

**DOI:** 10.1371/journal.pone.0080343

**Published:** 2013-11-11

**Authors:** Charlotte Gryseels, Sambunny Uk, Annette Erhart, René Gerrets, Vincent Sluydts, Lies Durnez, Joan Muela Ribera, Susanna Hausmann Muela, Didier Menard, Somony Heng, Tho Sochantha, Umberto D’Alessandro, Marc Coosemans, Koen Peeters Grietens

**Affiliations:** 1 Institute of Tropical Medicine, Antwerp, Belgium; 2 National Center for Parasitology, Entomology and Malaria Control, Phnom Penh, Cambodia; 3 The Amsterdam Institute for Social Science Research, University of Amsterdam, Amsterdam, The Netherlands; 4 Partners for Applied Social Sciences (PASS) International, Tessenderlo, Belgium; 5 Institut Pasteur du Cambodge, Phnom Penh, Cambodia; 6 Medical Research Council, Fajara, The Gambia; 7 University of Antwerp, Antwerp, Belgium; Centro de Pesquisa Rene Rachou/Fundação Oswaldo Cruz (Fiocruz-Minas), Brazil

## Abstract

**Background:**

Adherence to effective malaria medication is extremely important in the context of Cambodia’s elimination targets and drug resistance containment. Although the public sector health facilities are accessible to the local ethnic minorities of Ratanakiri province (Northeast Cambodia), their illness itineraries often lead them to private pharmacies selling “cocktails” and artemether injections, or to local diviners prescribing animal sacrifices to appease the spirits.

**Methods:**

The research design consisted of a mixed methods study, combining qualitative (in-depth interviews and participant observation) and quantitative methods (household and cross-sectional survey).

**Results:**

Three broad options for malaria treatment were identified: i) the public sector; ii) the private sector; iii) traditional treatment based on divination and ceremonial sacrifice. Treatment choice was influenced by the availability of treatment and provider, perceived side effects and efficacy of treatments, perceived etiology of symptoms, and patient-health provider encounters. Moreover, treatment paths proved to be highly flexible, changing mostly in relation to the perceived efficacy of a chosen treatment.

**Conclusions:**

Despite good availability of anti-malarial treatment in the public health sector, attendance remained low due to both structural and human behavioral factors. The common use and under-dosage of anti-malaria monotherapy in the private sector (single-dose injections, single-day drug cocktails) represents a threat not only for individual case management, but also for the regional plan of drug resistance containment and malaria elimination.

## Introduction

By scaling up malaria interventions and containing artemisinin resistance in the country, the Cambodian Government is currently trying to move towards phased elimination of malaria by 2025 [1]. The interest of donors, such as the Global Fund to fight AIDS, Tuberculosis and Malaria, the Bill and Melinda Gates Foundation and the Clinton Foundation, to invest in Cambodia has been largely fueled by events occurring along the Thai-Cambodian border, where *P. falciparum* antimalarial drug resistance first emerged [2]. The large-scale mining activities and the influx of migrant workers, combined with mass prophylactic monotherapy and/or substandard drugs sales of non-curative doses probably contributed to the emergence of chloroquine resistance in the town of Pailin almost half a century ago [2]. Recently, reduced sensitivity of *P. falciparum* to artemisinin – the last stronghold in malaria treatment – has been observed in the same area [3,4]. 

Effective malaria treatment is the cornerstone of successful malaria control, and artemisinin-based combination therapies (ACTs) are currently the most efficacious therapeutic option available. In Cambodia, ACTs are mainly available through community health centers (HCs) and volunteer Village Malaria Workers (VMWs) after diagnosis by Rapid Diagnostic Tests (RDTs). Therefore, good quality antimalarial treatment should be easily accessible. Nevertheless, poor treatment adherence could seriously hamper malaria elimination efforts as incomplete treatment would fail to clear infection and favor the spread of drug resistant parasites [5–7]. Therefore, the effectiveness of ACTs strongly depends on human behaviour [7], which is influenced by contextual factors and by people’s practical reasoning. In a context of malaria pre-elimination, these factors become increasingly important as each untreated or mistreated case could contribute to maintaining transmission [8,9]. This is even more relevant where malaria transmission occurs in remote forested areas due to the sylvatic and highly anthropophylic nature of the main vector *Anopheles dirus* [10,11], as is the case in Southeast Asia. These areas are often inhabited by poor and vulnerable ethnic minorities living off slash and burn agriculture, with substantial population movements within and across international borders. They often escape standard malaria control measures as these have been conceived for the largest section of the population and not for ethnic minorities with specific socio-cultural characteristics. 

In Cambodia, there is a preference for private healthcare and therefore the potential for an incorrect use of antimalarials [12]. In this context, ‘drug cocktails’ – provider-composed bags of mixed drugs – are extremely popular but hamper malaria elimination efforts by providing incomplete, substandard or inappropriate (monotherapy) treatment that can also be harmful [13–15]. It is therefore important to understand this phenomenon so that targeted control measures for elimination goals and the confinement of parasite resistance could be adjusted to this specific context. 

## Methods

### Study site and population

#### Population

The study was carried out in the Cambodian province of Ratanakiri, which in 2008 had a population of 150,000 inhabitants (1% of the total Cambodian population) [16]. The study villages were home to 10 indigenous ethnic groups [17], the Jarai, the Tompuon and the Kreung being the largest of them. Most ethnic minorities practice slash and burn agriculture at forest fields and collect various forest products (hunting and gathering). They often live where they are working, and as such may have several residences spread over the village, rice field or forest farm. Animistic beliefs require agricultural activities to be performed in cooperation with the spirits and ancestors that pervade the trees, rocks and rivers of the village territory [18–20]. 

#### Malaria transmission

Malaria transmission is perennial with two peaks, June-July and October-November, the rainy season lasting from May to October. The main malaria vector is *Anopheles dirus*, an anthropophylic, outdoor and sylvatic species [10,11]. *P. falciparum* and *P. vivax* prevalence is similar and in 2010 the overall incidence of clinical malaria was 49/1000 population [21]. Given the high number of Long Lasting Insecticidal Nets (LLINs) distributed by the National Malaria Control Program (1 net per 2 persons since 2008 and 1 net per person since 2011), malaria transmission is assumed to occur mostly outdoors, and before or after sleeping time, when people are not protected by bed nets [11,22]. 

#### Malaria treatment guidelines

In the public sector, malaria control measures are implemented through VMWs at community level, health posts at commune level, health centres at district level and referral hospitals usually in the provincial capital. At the time of this study, the first-line treatment was prepackaged (blister pack) mefloquine-artesunate for *P. falciparum* and chloroquine for *P. vivax* - both taken over three days. Mefloquine-artesunate, provided under the brand name A+M, is officially available free of charge at the commune health posts, district health centers and the provincial hospital. For severe malaria, the recommended treatment was either artemether or quinine injections, available at the provincial referral hospital and certain district health centers. 

In remote villages, VMWs, after confirmation by an RDT, can administer A+M free of charge [15,23,24]. They are supposed to keep detailed records of both RDT results and treatments given and are supervised by the district health centre staff that check the records and replenish their stock. 

**Figure 1:**
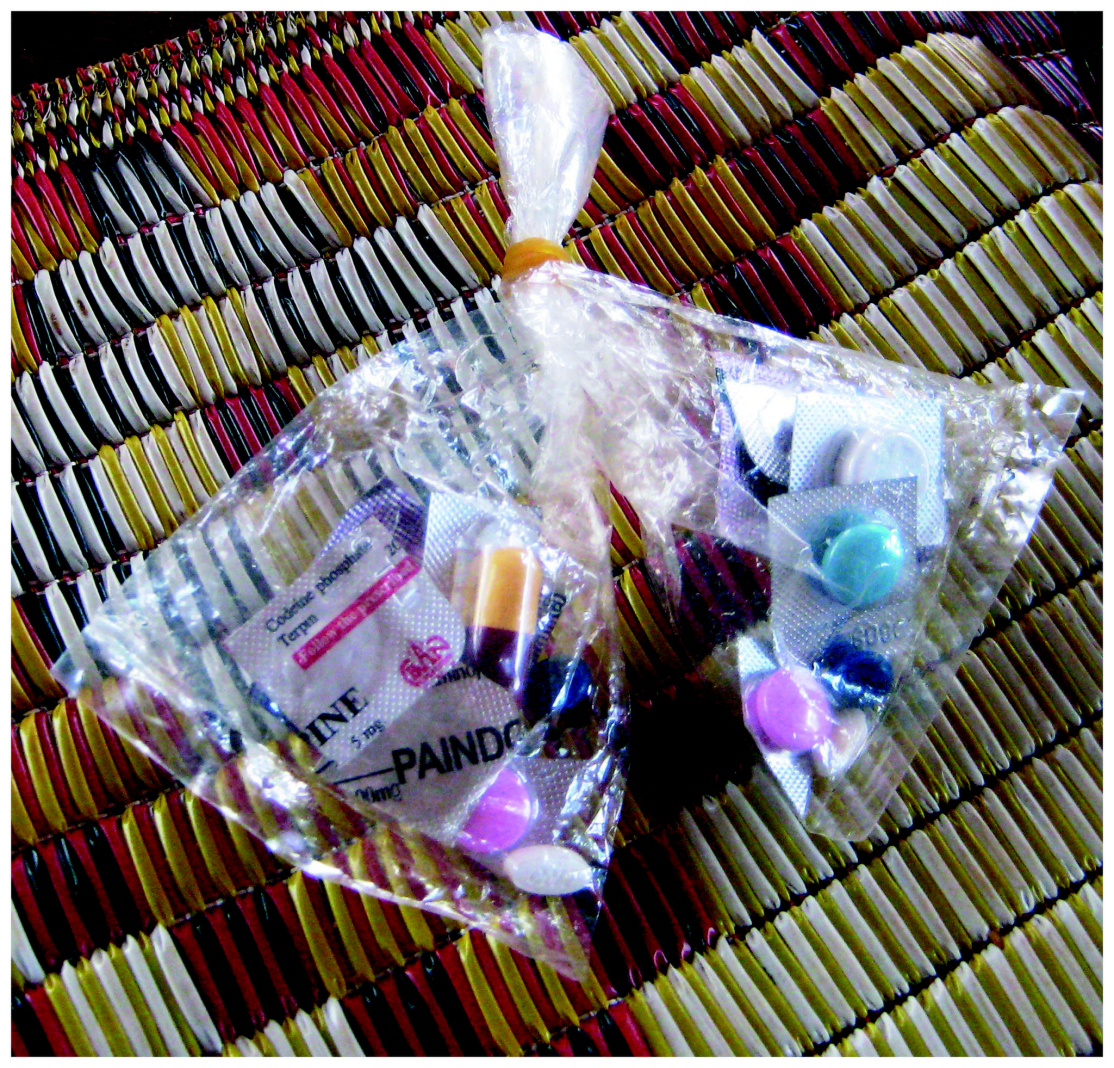
Local “anti-malaria cocktail”.

The private sector in Cambodia is officially organized in four levels of recognized practitioners: (i) private clinics run by medical doctors, (ii) pharmacies with a registered pharmacist, (iii) ‘Depot A’ pharmacies run by assistant pharmacists and (iii) ‘Depot B’ pharmacies operated by nurses or midwifes [25]. In terms of antimalarial treatment, Population Services International (PSI) provides Malarine™ blisterpacks (mefloquine-artesunate) to the private sector, which is sold at highly subsidized prizes. Nevertheless, in addition, private practitioners or pharmacists can assemble “cocktails” for their clients [13,15]. One cocktail consists of a one-day treatment dose combining pills cut off from a blister pack strip or taken from a jar, and provided in a little plastic bag (see Figure 1). 

In addition, the ethnic minority groups in Ratanakiri consult diviners, i.e. traditional healers able to spiritually divine the cause of an illness. Research on home treatment such as coin massages and the use of herbs is outside the scope of this study and was not further explored. 

### Research strategy

The research was based on a triangulation design, combining qualitative and quantitative data collection techniques in order to strengthen the validity of the data and to achieve complementarity, allowing the quantification of treatment options and treatment choice.

In a first phase of research, a mixed methods study design, consisting of qualitative ethnographic research and a household survey, was carried out in the Oyadao health district, a sub-setting of the study area. This represented an in-depth exploratory case study, aiming at gaining an in-depth understanding of the research questions and at testing preliminary hypotheses about the use of anti-malaria treatment. In a second research phase, complementary quantitative data were gathered from a large-scale cross-sectional survey. 

### Qualitative study

#### Data collection

Fieldwork was conducted between April and July in 2010 in Oyadao district, mostly in the border villages of Lom and Phi. For participant observation, the research team participated actively in and observed the daily life of the study population. Continuous informal conversations and interviews with respondents built up confidence for the discussions on more sensitive issues such as adherence to treatment obtained from public facilities. Frequent overnight stays in the two study villages, multiple visits to 11 private sector establishments (including pharmacies and private practitioners) along the main road in Oyadao district, and observation sessions at the health center and health post during consultation hours and monthly VMW meetings, were used to collect information on people’s behavior and to compare it with the information given by interviewees. This was an essential step to uncover response bias.

A total of 126 interviews were carried out with various respondents from different social, ethnic, professional and economic categories. These interviews were recorded and transcribed. When a conversation was of a more informal nature, or when the interviewer decided it was inappropriate to record the interview or the interviewee refused recording, notes were taken immediately after the interview. An additional 32 unrecorded informal conversations were included in the analysis. 

#### Sampling

Respondents were theoretically selected, referring to the purposive, gradual and continuous inclusion of respondents based on emerging results during the research process. Access to respondents was usually granted through snowball-sampling techniques, where certain key-informants introduce the researcher to selected participants. Many informants were visited several times as an additional way of building confidence between researcher and respondent, hence reducing bias.

### Quantitative study

#### Data collection

After the qualitative strand of the study, two surveys were carried out. In the household survey a structured questionnaire was administered to all household leaders of the two villages involved in the qualitative study. Questions focused on the different providers of antimalarial treatment and on the choice for their last malaria episode (health centre, private practitioners, VMWs, traditional healers, etc.), including the reason for the choice. The other survey, a large scale cross-sectional survey, focused exclusively on treatment choice. 

#### Sampling

For the household survey, all households in the two study villages for the qualitative study (Phi and Lom) were sampled. For the cross-sectional survey, 900 individuals were randomly selected from the census of the villages included in the research project “Repellents as an Added Control Measure to Long-lasting Insecticidal Nets in Southeast Asia”, a community-based trial implemented in 113 villages of Ratanakiri province. The sample size was calculated based on the expected difference between study arms in the main outcome variable of the overall study, which was repellent use in villages and at farms. In total, 824 people from 109 different villages answered the structured questionnaire. An additional non-response form was used to measure possible systematic self-selection bias.

### Data analysis

#### Qualitative data

Thematic analysis was continuously carried out, after which emerging results and hypotheses were tested in the field until saturation was reached. For the preparation of the study and for analytical purposes, the PASS Health Seeking Behaviour Model was used [26]. Briefly, this model presents various elements that guide health seeking behaviour and access to care and, as such, can be adapted to different research contexts. The final analysis of all qualitative data was carried out in NVivo 9 Qualitative Analysis Software.

#### Quantitative data

Preliminary analysis of the qualitative data was used to build the standardized questionnaires for the two quantitative surveys. Quantitative data were entered in Excel and analysed in SPSS (IBM SPSS Statistics 19). Frequency tables for the main outcome variables were produced, i.e. perceived VMW treatment availability, availability during last visit to VMW, treatment taken during last malaria episode, provider visited during last malaria episode. Data on the perceived performance of the VMWs were elicited only in the household survey (real life, non-trial setting). 

### Ethical considerations

The study protocol was approved by the National Ethics Committee for Health Research in Cambodia and The Institutional Review Board of ITM, Antwerp. The interviewers followed the Code of Ethics of the American Anthropological Association (AAA). As proposed by the AAA, all interviewees were informed before the start of the interview about project goals, the topic and type of questions, the intended use of results for scientific publications as well as their right to reject being interviewed, to interrupt the conversation at any time, and to withdraw any given information during or after the interview. Anonymity was guaranteed and confidentiality of interviewees assured by assigning a unique code number to each informant. The interviewers sought oral consent from all interviewees. Oral consent was preferable, since the act of signing one's name when providing certain information can be considered a potential reason for mistrust. Both ethics committees approved the verbal consent procedure.

## Results

### Choice of public or private health providers

#### Village Malaria Workers

More than half (55.7%) of all household survey participants reported having ever visited their VMW, and 54.0% of those who had had malaria in the past reported having received treatment from the VMW for their last malaria episode (Table 1). However, qualitative interviews with villagers and VMWs revealed the general perception that the VMW’s ran out of stock (RDTs/treatments) rapidly during the rainy season, either due to the difficulty to replenish their stock (heavy workload in the fields, inaccessibility) or due to a higher demand. Indeed, in the household survey, 50.8% of all survey respondents perceived the VMW did not always have medication, 60.6% did not think the VMW would have the required test available, and 48% reported that the VMW was not always available when visited by patients. When asked about their last visit to the VMW, 30.7% of the respondents answered that the VMW did not have a test available, and 36.5% that there was treatment stock out. (Table 1). 

**Table 1:** Household survey in Phi and Lom villages.

**General perception of VMW treatment (n=246)**	**N**	**%**
Ever visited the VMW when suspected to have malaria:		
*- Never suspected to have malaria*	76	30,9
*- Ever visited the VMW*	137	55,7
*- Never visited the VMW*	32	13,0
Perception of VMW treatment availability:		
- *VMW* ** *does* ** *not* ** *always* ** *have* ** *enough* ** *tests* ** *available*	149	60,6
*- VMW does not always have enough medication available*	125	50,8
*- VMW is not always available*	118	48,0
**Treatment options during last malaria episode (n=161)**	**N**	**%**
Where did you buy treatment?		
- *Health* ** *center*	15	9,3
*- Hospital*	2	1,2
*- Pharmacy*	26	16,1
*- Private doctor*	12	7,5
*- VMW*	86	54,0
*- Market*	17	10,6
*- Other*	1	0,6
*- Missing*	2	1,2
Type of treatment taken during last malaria episode?		
- *Pills* ** *- cocktails*	22	13,7
*- Pills - blisterpacks*	126	78,3
*- Injections*	10	6,2
**Treatment availability during last visit to the VMW (n=137)**	**N**	**%**
Test was not available	42	30,7
Medication was not available	50	36,5
VMW was not available	21	15,3

#### Health centres, health posts and hospitals

Only 9.3% of respondents in the household survey had attended the health centre during their last suspected malaria episode, and 1.2% had attended the provincial referral hospital (Table 1). Qualitative data consistently showed that only severe cases attended or were referred to the hospital by health centre staff or VMWs. 

#### Private sector

The following categories of private health providers were identified through observation and in-depth interviews with villagers, health staff and private practitioners: (i) *medical cabinets*, referring to private practitioners, not necessarily with any official medical training, working in a medical cabinet; (ii) *pharmacies*: practitioners (sometimes nurses from the health center, sometimes people without any medical training or untrained pharmacists) owning pharmacies and providing treatment; (iii) *mobile private practitioners*: practitioners visiting villages when called upon by patients; (iv) *informal drug selling* (grocery shops or individuals). In the household survey, 34.2% of respondents reported having attended one of private sector options during their last malaria episode (private practitioners, pharmacies, market) (Table 1). 

#### Public-private overlap

Despite the general distinction between public and private sectors, qualitative data clearly showed that this distinction is not so clear-cut. Indeed, each of the health center staff interviewed or observed owned his/her own private pharmacy at home, which was usually located near the health center. During informal conversations at health centers, informants reported that the medical staff often ‘advised’ people to buy the medication at their private pharmacy before being treated at the health centre, particularly when the type of medication desired was not available at the health center (e.g. artemether injections). 

### Choice of public or private sector treatment (Figure 2)

**Figure 2:**
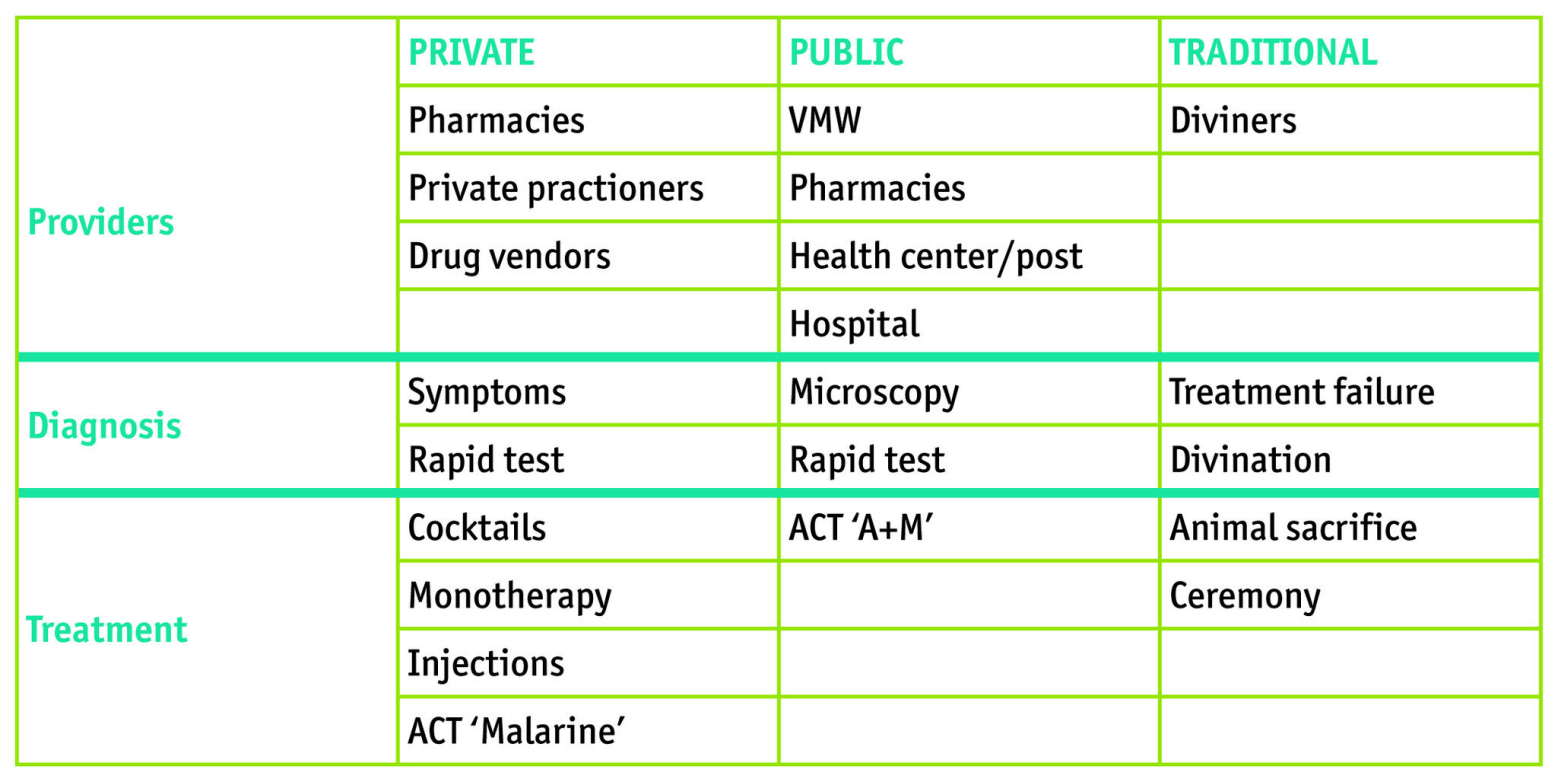
Model for Treatment Options.

#### Blister packs

Observations and interviews indicate that blister packs were common in both public and private sectors. In both the household and cross-sectional surveys, more than 75% of the respondents claimed to have bought pills in blister packs during the last malaria episode (Table 1 and 2). 

**Table 2:** Cross-sectional survey.

**Treatment taken during last malaria episode of a household member (n=711)**	**N**	**%**
No treatment taken	4	0,5
Treatments separate		
- *Injections*	229	32,2
*- Infusions*	188	26,4
*- Pills - Blisterpack*	555	78,1
*- Pills - Cocktails*	185	26,0
*- Animal sacrifice*	265	37,3
*- Herbal treatment*	103	14,5
*- Coin massage*	285	40,1
Treatments combined		
*- Substandard biomedical treatment exclusively (injections, infusions, cocktails*)	59	8,3
- *Standard biomedical treatment exclusively (blisterpacks*)	141	19,8
*- Traditional treatment exclusively (sacrifice, herbal, coin massage*)	1	0,1
*- Substandard, standard and traditional treatment combined*	119	16,7
*- Standard and traditional treatment combined*	232	32,6
*- Substandard and traditional treatment combined*	92	12,9
*- Standard and substandard treatment combined*	63	8,9
Number of treatments combined		
*- 1*	157	22,1
*- 2*	217	30,5
*- 3*	180	25,3
*- 4*	98	13,8
*- 5*	40	5,6
*- 6*	11	1,5
*- 7*	3	0,4

#### Cocktails

Following repeated observations at private facilities as well as respondents’ homes, a cocktail to treat malaria contained one or several antipyretic/analgesics (paracetamol), one antibiotic, vitamins, and one kind of antimalarial monotherapy (either chloroquine or artesunate) (Figure 1). Informants reported to buy one cocktail for around zero point two fiveUS$, and usually bought as many as they could afford. 13.7% of respondents in the household survey reported having bought cocktails for their last malaria episode, while this percentage was 26.0% in the cross-sectional survey (Table 1 and 2). 

#### Injections

Observations in pharmacies and interviews with informants indicate that artemether injections (intra-muscular), both European and Asian brands, were for sale in most of the private sector establishments, and were usually administered by the private practitioner or, in order to reduce the cost, were bought as ‘take-away’ for self-administration. Private practitioners reported the latter option to be the most popular since patients could often not afford to buy a full-dose 5-day injection treatment. In the household survey, 6.2% of household leaders reported having bought injections during their last malaria infection (Table 1), while this was 32.2% in the cross-sectional survey (Table 2). 

#### Infusions

Although generally not perceived as a malaria treatment, respondents in the qualitative study often reported intravenous infusions (glucose/vitamins) to be necessary to relieve symptoms and ‘strengthen’ the patient during a malaria episode. According to the cross-sectional survey, 26.4% of the respondents stated to have bought infusions during the last malaria episode for one of their household members.

### Choice of traditional health providers and treatment (Figure 2)

#### Diviners

In the qualitative study area, we identified two diviners (specialized villagers that can divine the causes of illness and discomforts for a small fee). Informants from those villages stated that it was common for each village to have at least one diviner. Interviews with both villagers and diviners showed that illness etiologies pointed out by diviners referred to the transgression of social norms by the patient, and sacrificial actions to restore health. The diagnosed transgressions usually involved a social disruption (familial disputes, failure to perform ceremonies). Treatments of patients mostly consisted of a ceremony (a social event involving the drinking of rice wine) and sacrifice of a costly buffalo, pig or chicken to the involved spirit, ancestor or witch, depending on the nature of the transgression and the financial situation of the family. In the cross-sectional survey, 37.3% of respondents claimed to have performed a ceremonial animal sacrifice during the last malaria episode of one of the household’s members, although most of them combined this treatment with “modern medicine” (blister pack, injections or cocktails).

### Determinants for treatment choice

While the household survey showed that the large majority of respondents turned to the VMW for suspected malaria, the qualitative research revealed a preference for general treatment outside the public health sector for the following reasons.

#### Availability of treatment

According to interviews and informal conversations with villagers, the VMW is usually the first option for suspected malaria but if he is not available or does not have treatment or diagnostic tests, people are reluctant to travel to the health centre. 

#### Perceived side effects

Both A+M and Malarine™ were perceived to cause strong side effects, i.e. dizziness and vomiting, and were therefore avoided whenever possible, as illustrated by the following quote: “My husband also got medication from the VMW. He took it for three days, but it had very strong side effects. He almost committed suicide!” (Jarai farmer, Phi). The mentioned side effects were one of the factors leading people to seek treatment in the private sector, either to buy antimalarial cocktails or injections, or to reduce the adverse effects of the public sector treatment with infusions, cocktails or injections. Some patients report to ‘preventively’ get an infusion at the private practitioner to counteract the expected side effects of the first-line ACT they would get from the VMW or HC staff. Although most pharmacies advertised for Malarine™, private health providers often reported that patients able to afford artemether injections chose for this more expensive but better tolerated option. 

#### Perceived efficacy of the drug

Despite the official indication for hospital use only, qualitative research showed that artemether injections were available in pharmacies without medical prescription. Reasons for their popularity were the quick relief provided which, together with the absence of side effects, allowed patients to rapidly return to their fields, where the workload is the highest during the malaria (rainy) season. Injections were also preferred because they would avoid swallowing of pills by an already weak patient, an often disliked act. 

#### Expected costs

Villagers reported prices ranging from 0.35 to 5 US$ for a consultation at the HC. Prices were similar for private practitioners but without the long waiting period, an appealing option that reduced the loss of productivity. Given the affordable price of cocktails compared to injections (0.25$ versus 2.5$), the former were quite popular and patients were usually able to buy 2-3 single-dose bags. 

#### Health provider - patient encounter

Patients’ reluctance to consult the local HC was not only related to the long waiting hours, but also by the fact that HC staff did not have the time to attend the numerous patients waiting for treatment. Informants often reported on the ‘unfriendly’ reception by the HC personnel, stressing the inherent hierarchy between doctor and patient, and sometimes between Khmer and ethnic minority. Conversely, private practitioners were reported to ‘take better care’ of their patients, taking more time, and delivering instant care with rapid tests and the client’s choice of medication. 

#### Perceived etiology

Despite the overall good knowledge of the “cause-symptoms-treatment” relations of malaria in the study area, the same symptoms could also be perceived as having a “supernatural” etiology, as illustrated by the following quote: “My children took malaria medication and recovered. But for me it was necessary to make an offer. I took the medication for only one day, and almost died. [So] I have a different problem: with the spirits. After one day of medication I offered a pig to the spirits. Before we go to the VMW, we go to meet the diviner and we follow the diviner” (Jarai farmer, Phi village). Thus, it was not uncommon that villagers experiencing symptoms such as fever, chills and headaches, consulted immediately the local diviner before considering biomedical treatment options. 

Moreover, although illnesses of natural origin and those of supernatural origin were not seen as directly related, they could interact and aggravate the patient’s situation as illustrated hereafter: “The witch makes sure you don’t get hungry, you become skinny, or she makes you have pain all over your body. If this is combined with malaria, you die even sooner” (Jarai farmer, Phi village). 

### Flexibility of treatment paths (Figure 3)

**Figure 3:**
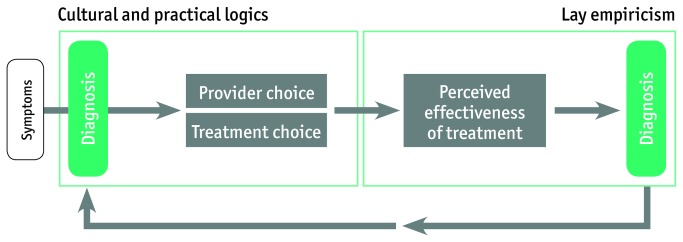
Model for Therapeutic Flexibility.

Although many patients first consult the VMW or private sector to find “modern medicine”, it was, however, not uncommon that people perceived that the medication did not work well. When patients did not feel cured after taking anti-malarials, alternative options were explored, including the diviners. Similarly, the diviner’s knowledge was not considered flawless either, leading to a highly flexible treatment path in which treatment choice was adjusted according to the need of the patient, its economic situation, and the perceived efficacy of the treatment. This is illustrated by the following quote: “Last time I got ill, I went to the private practitioner to get an injection or infusion, then I left to Ban Lung for more treatment, and then I went to Vietnam, but I still did not recover. So after visiting three places I was still not better, so then I came back to the village and asked the diviner. The diviner said that I had to sacrifice a buffalo. So I bought the buffalo […] and after that I did recover” (Jarai farmer, Phi Village). This flexibility implies that the perceived etiology is related to the perceived efficacy of the first or prior treatment(s), making the latter a form of diagnosis based on lay empiricism. As the results from the cross-sectional survey indicate, treating exclusively with one type of treatment is not common: 77,1% combined two or more treatments during the last malaria episode of a household member.

## Discussion

Despite good accessibility of public health facilities, the latter were not always the preferred treatment choice for malaria in rural and remote Ratanakiri. Although each village had a VMW, who was often the first step on the treatment pathway, the community perception that the VMW was not readily available, especially during the malaria season, was one of the reasons why people turned to the private sector and/or traditional treatment. An additional factor driving patients to the private health care providers was the perceived side effects of the first line treatment A+M (or Malarine™). ACT with mefloquine as partner drug is well known to be associated with stronger side effects (acute nausea, vomiting, anorexia and dizziness) compared to other ACTs or artemisinin-derived monotherapies [27], providing evidence for people’s fear of A+M’s side effects and their preference for artemether injections and cocktails. 

Despite the methodological differences as well as different variables being measured between the household and cross-sectional surveys, both of them estimated that approximately one third of the respondents chose private sector treatments. The use of private sector treatment alternatives - injections and cocktails - has economic consequences since families may spend their savings on several suboptimal treatments, while they might have been cured by one complete ACT course. Although cocktails are a cheap option, they are not economically sound as several doses must be bought over time or additional and different medication has to be purchased at a later stage. This finding was also confirmed for Cambodia by Trankell and Ovesen: “one unfortunate effect of this cultural expectation is that the small amount of money most customers have at their disposal will be spread over many non-essential products” [13]. Moreover, the perceived treatment failure and the recurrence of symptoms can be related to such systematic under-dosage of anti-malaria monotherapy in the private sector (single-dose injections, single-day drug cocktails). This is even more worrying when knowing sub-standard and fake drugs are widely circulating on local markets in Southeast Asia [6,28]. 

A consequence of the perceived lack of efficacy and treatment failure of ‘modern medicine’ - be it cocktails, injections or blisterpacks - is that people turn to traditional medical systems. This has been shown in Tanzania, where witchcraft interpretations of illness are logical in the context of biomedical treatment failure and the overemphasis of the infallibility of biomedical treatment by educational health messages [29]. The perceived treatment failure encourages people to search for supernatural explanations for their illnesses. As such, the empirical evaluation of the efficacy of the treatment leads patients to search for a different cause of their symptoms, in other words the perceived treatment failure becomes a diagnostic tool [30]. 

The mentioned therapeutic pathways clearly show the interaction between and the complementarity of the different treatments, indicating highly flexible itineraries instead of a static sequence of health-seeking actions. This variation and flexibility is based on a form of lay empiricism rooted in local cultural logics and perceived treatment efficacy. First, previous experience provides the basis for the rationale (or practical reasoning) of the treatment choice (for example, being able to go back to work after taking a cocktail) while perceived treatment efficacy provides a diagnostic tool for further therapeutic guidance.

Although the preference for these biomedically ‘irrational’ practices is indeed logical within this social context, the situation nonetheless may have serious consequences for malaria elimination goals, as these mistreated (private sector) or non-treated (traditional treatment) cases result in a parasite reservoir that can maintain transmission, even where the malaria burden is decreased substantially thanks to the large scale implementation of control interventions. Moreover, the popularity of cocktails and the private sector’s potential to deliver these in the form of single-day doses of monotherapies can have a disastrous effect as it would increase the selective drug pressure and favor the spread of resistant parasites [31]. The situation observed in Ratanakiri occurs in other parts of Cambodia [14] and probably in other areas of the Greater Mekong Sub-region. This is why it is plausible that, although a program for the containment of artemisinin resistance is implemented on the Thai-Cambodian border, other foci of artemisinin resistance may appear elsewhere. Indeed, delayed parasite clearance has been reported in the Vietnamese border provinces of Binh Phuoc and, more recently, Quang Nam [32,33]. 

## Conclusion

The use and/or under-dosage of anti-malaria monotherapy in the private sector (injections, drug cocktails) represents a threat, not only for the individual patient (i.e. recurrences and aggravation of symptoms) but also for the community as the resulting drug pressure can favor the spread of resistant parasites [31]. This is an even greater concern when considering that sub-standard and fake drugs are widely available in local private establishments in Cambodia [6,28]. Local behavioral and contextual factors need to be taken into account when designing interventions aiming at containing artemisinin resistance and decreasing the malaria burden in ethnic minorities living at the edges of mainstream society. Determinants for treatment choice, such as perceived etiology, perceived side effects, perceived efficacy of the drug, expected costs, attitudes and behaviors of the health staff should receive increased attention when engaging health and non-health sectors to reach high-risk populations. This paper advocates for an inclusion of such socio-cultural determinants into the regional framework for action of the emergency response to artemisinin resistance in the Greater Mekong subregion [34]. 
